# Knowledge Management in Orthopedic Hospitals: A Narrative Review of Digital Infrastructure, Multidisciplinary Coordination, and Patient Safety

**DOI:** 10.7759/cureus.107764

**Published:** 2026-04-26

**Authors:** Xiao Du, Mingyuan Zhao, Zhuo Fu

**Affiliations:** 1 Department of Healthcare Quality and Safety, Luoyang Orthopedic-Traumatological Hospital of Henan Province (Henan Provincial Orthopedic Hospital), Luoyang, CHN; 2 Faculty of Business and Communications, INTI International University, Nilai, MYS; 3 Department of Medical Affairs, Luoyang Orthopedic-Traumatological Hospital of Henan Province (Henan Provincial Orthopedic Hospital), Luoyang, CHN; 4 Center for Medical Ultrasound, Luoyang Orthopedic-Traumatological Hospital of Henan Province (Henan Provincial Orthopedic Hospital), Luoyang, CHN

**Keywords:** clinical integration, digital health, evidence-based practice, health informatics, knowledge management, multidisciplinary care, orthopedic hospitals, patient safety

## Abstract

Orthopedic hospitals are knowledge-intensive settings shaped by high procedural volume, multidisciplinary coordination, implant- and imaging-based decision-making, and extended rehabilitation pathways. However, knowledge management (KM) in orthopedic healthcare remains insufficiently synthesized. This review examined current evidence on KM in orthopedic healthcare and proposed an orthopedic-specific framework linking KM processes with patient safety and organizational performance. A structured narrative review was conducted using PubMed/MEDLINE, Scopus, Web of Science, Embase, CINAHL, and Google Scholar. Literature published from January 2020 to March 2026 was prioritized, with selective inclusion of seminal theoretical, registry, and policy sources. Eligible studies addressed hospital-based orthopedic care or related musculoskeletal, surgical, nursing, and digital health contexts relevant to knowledge capture, sharing, evidence use, implementation, patient safety, and performance. Given the heterogeneity of the included literature, findings were synthesized thematically and interpreted in relation to study characteristics, practical relevance, contextual transferability, and conceptual contribution. A total of 88 sources were included. Six recurring themes were identified across orthopedic-specific and transferable healthcare literature: KM governance; knowledge capture and codification; multidisciplinary knowledge sharing; evidence use and pathway standardization; technology-enabled KM; and implementation barriers and enablers. Orthopedic hospitals showed distinctive KM needs related to implant surveillance, perioperative coordination, rehabilitation continuity, outcome feedback, and tacit procedural expertise. This review suggests that KM may be regarded as a core component of clinical infrastructure in orthopedic healthcare. Strengthening KM may support evidence-based practice, multidisciplinary coordination, digital capability, and patient safety. Future research should prioritize orthopedic-specific KM metrics and prospective evaluation of human-centered and digital integration strategies.

## Introduction and background

Knowledge management (KM) has become pivotal to contemporary healthcare delivery, as modern hospitals increasingly rely on the rapid creation, storage, dissemination, interpretation, and application of knowledge under considerable time constraints [1-4]. This imperative is particularly pronounced within orthopedic hospitals. Orthopedic care is inherently multifaceted, integrating elective and emergent interventions, high-volume surgical procedures, implant-intensive practices, imaging-dependent diagnostics, comprehensive perioperative risk management, and extended rehabilitation trajectories [5-9]. Consequently, clinical workflows are driven by a complex synthesis of tacit experiential knowledge, formal clinical guidelines, device-generated data, patient-reported metrics, registry surveillance, and continuous interdisciplinary coordination across wards, operating theaters, outpatient clinics, and rehabilitation facilities [5,6,10-14]. When these vital knowledge streams remain fragmented, healthcare institutions become susceptible to unwarranted practice variations, delayed organizational learning, documentation deficiencies, compromised care continuity, and preventable safety hazards [15-20].

Recent scholarship in healthcare KM offers a valuable framework connecting broader KM theories to the operational realities of orthopedic institutions [2,21-24]. Existing research consistently demonstrates that the effective generation, sharing, and utilization of knowledge significantly correlate with enhanced evidence-based practice, superior service quality, elevated workforce performance, and augmented managerial efficacy [24-31]. Nevertheless, this body of literature also reveals a persistent research gap: the field remains largely saturated with either generalized healthcare analyses or strictly technology-centric investigations, leaving the dynamic interplay among human behavior, intricate health systems, and KM technologies insufficiently explored [2,21-23,28,29]. Orthopedic hospitals epitomize this intersection. While they operate as highly technical environments, their success simultaneously relies on robust interdisciplinary teamwork, clinical judgment, and institutional memory [15,19,20,30-33].

A dedicated review of KM within the context of orthopedics is timely for several reasons [5-7,34-36]. First, orthopedic decision-making is exceptionally multidimensional, routinely synthesizing diagnostic imaging, clinical symptomatology, patient preferences, surgical experience, implant familiarity, and frequently ambiguous empirical evidence [34,35]. Recent investigations into practice variations within orthopedics indicate that formal guidelines do not intrinsically supplant experiential or localized knowledge [34,37,38]. Second, orthopedic facilities increasingly depend on clinical registries, performance dashboards, patient-reported outcome measures (PROMs), infection surveillance systems, and implant traceability frameworks, all of which function as critical KM assets when effectively integrated [5,6,10-14,39-42]. Third, complex perioperative pathways, such as those for arthroplasty and fracture management, necessitate seamless knowledge transfer among diverse professionals, including surgeons, anesthesiologists, nursing staff, physiotherapists, pharmacists, and discharge coordinators [16-20,43,44]. Fourth, because orthopedic clinical outcomes evolve over extended periods, institutional learning is required to transcend traditional organizational boundaries, bridging inpatient wards, outpatient rehabilitation, and community-based care [10-14,39,45]. Finally, the expanding use of artificial intelligence (AI), telehealth modalities, and digital documentation systems may offer important opportunities, although their benefits are likely to depend on how well foundational clinical knowledge is systematically curated, accurately interpreted, and trusted [7,46-51]. Accordingly, this review aimed to synthesize and interpret the literature on KM in orthopedic hospital settings, while examining how KM relates to evidence use, multidisciplinary coordination, digital infrastructure, patient safety, and organizational performance.

## Review

Methodology

Search Strategy and Information Sources

This article presents a structured narrative review utilizing transparent literature search and selection protocols. The methodological design adheres to the core principles of narrative and mapping reviews while retaining the flexibility required to integrate diverse paradigms of evidence. Accordingly, instead of generating pooled effect estimates, the primary objective was to synthesize and interpret a heterogeneous body of literature concerning KM in orthopedic hospital settings, including its relationship to evidence use, knowledge sharing, digital systems, patient safety, and organizational performance.

A comprehensive literature search was conducted across several major electronic databases, including PubMed/Medical Literature Analysis and Retrieval System Online (MEDLINE), Scopus, Web of Science, Excerpta Medica database (Embase), Cumulative Index to Nursing and Allied Health Literature (CINAHL), and Google Scholar. To ensure the review encapsulates contemporary advancements, the primary search window spanned from January 2020 to March 2026. The searches were conducted on 23 March 2026 and last updated on 27 March 2026. The full database-specific search strategies are provided in the Appendices. Nevertheless, earlier seminal studies, theoretical papers, and classic references pertaining to implementation or clinical registries were purposively incorporated when necessary to establish a foundational context for key concepts, definitions, and institutional developments. Search queries were formulated by synthesizing terms related to knowledge management (“knowledge management”, “knowledge sharing”, “knowledge transfer”, “knowledge mobilization”, “knowledge translation”, “organizational learning”, “clinical decision support”, “evidence-based practice”), orthopedic care (“orthopedic”, “orthopaedic”, “trauma”, “arthroplasty”, “musculoskeletal”, “joint replacement”, “fracture”), hospital settings (“hospital”, “surgical ward”, “operating room”, “rehabilitation”, “acute care”), and digital or organizational enablers (“electronic health record”, “registry”, “dashboard”, “PROM”, “artificial intelligence”, “implementation”, “multidisciplinary team”).

Eligibility Criteria

Eligible literature comprised a comprehensive range of sources, including peer-reviewed journal articles, systematic, scoping, and narrative reviews, mixed-methods studies, qualitative and quantitative observational research, implementation studies, selected consensus or policy documents, and landmark registry reports. Articles were deemed eligible for inclusion if they investigated at least one of the following core domains: hospital-based KM within healthcare; orthopedic decision-making or practice variations; digital systems relevant to knowledge capture or application; the implementation of evidence-based practice; multidisciplinary coordination; competency development; patient safety; clinical registries; PROM utilization; or related implementation strategies within surgical or musculoskeletal contexts. Priority was accorded to studies demonstrating clear relevance to hospital or specialty service delivery. For synthesis purposes, included sources were interpreted as either orthopedic-specific literature (directly focused on orthopedic hospitals, orthopedic services, or musculoskeletal surgical pathways) or transferable literature (from related hospital-based healthcare settings with KM mechanisms judged applicable to orthopedic practice). Conversely, commentaries lacking substantive analysis, duplicate publications, and manuscripts without demonstrable applicability to KM in orthopedic hospital settings were excluded.

Screening and Selection

**Figure 1:**
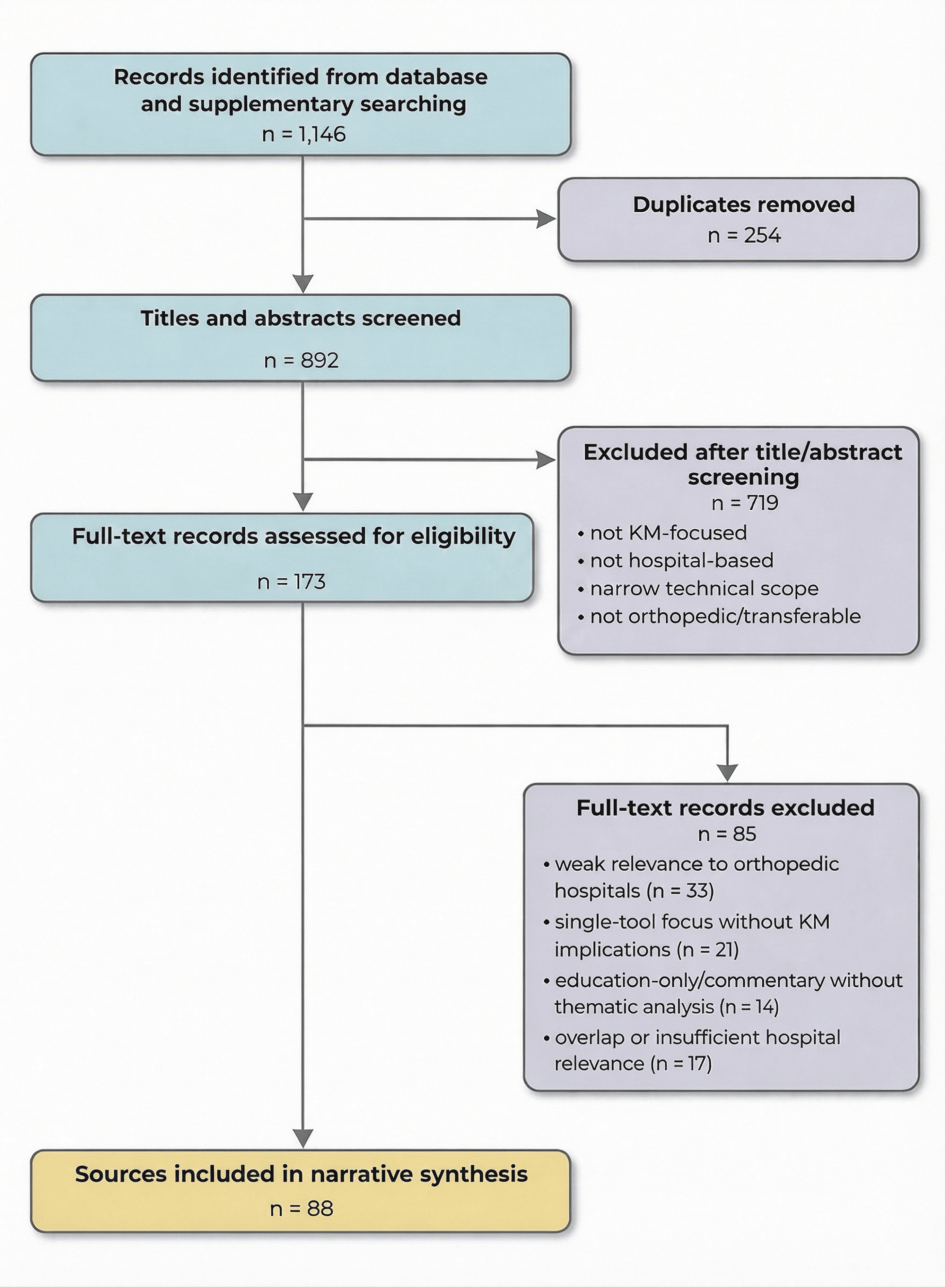
PRISMA-adapted study selection flow diagram KM: knowledge management; PRISMA: Preferred Reporting Items for Systematic Reviews and Meta-Analyses.

Initial database searches identified a total of 1146 records. Following the removal of 254 duplicates, 892 titles and abstracts underwent primary screening against the established eligibility criteria. Title/abstract screening and full-text assessment were conducted by two reviewers (X.D. and M.Z.). These assessments were performed independently, and disagreements were resolved through discussion and consultation with the third reviewer (Z.F.). Subsequently, 719 records were excluded due to a lack of relevance to KM, an absence of hospital focus, a purely technical nature without organizational implications, or because they fell outside orthopedic or plausibly transferable healthcare contexts. Full-text evaluations for eligibility were then conducted for the remaining 173 articles. Of these, 85 were excluded, predominantly because they centered on isolated digital tools without broader KM implications, addressed educational initiatives within overly narrow scopes, or lacked sufficient applicability to hospital settings or orthopedic care pathways. Moreover, given that orthopedic hospital KM is sparsely directly addressed in the existing literature, this review drew on recent cross-cutting studies encompassing healthcare KM, digital innovation, evidence-based practice, managerial competence, smart technology, and nursing-focused knowledge management system (KMS) design (Table 1). These sources were incorporated as contextually pertinent literature to better understand the dynamic interactions among technology, human behavior, and organizational conditions in hospital-based knowledge work. Furthermore, they provided transferable insights essential for identifying mechanisms, boundary conditions, and research gaps directly applicable to orthopedic institutions. In the synthesis, these studies were treated as transferable rather than direct orthopedic evidence and were analyzed with attention to contextual relevance and limits of transferability. Ultimately, 88 sources were included in the final narrative synthesis. For clarity, the included sources were interpreted broadly as orthopedic-specific studies, transferable general healthcare studies, and theoretical/policy/registry sources used for conceptual grounding. Figure 1 illustrates the structured search and screening process, presented via a PRISMA (Preferred Reporting Items for Systematic Reviews and Meta-Analyses)-adapted flow diagram to enhance methodological transparency.

**Table 1:** Selected recent cross-cutting healthcare KM studies informing the thematic scope of the review KM: knowledge management; AI: artificial intelligence; SEM: structural equation modeling; EBP: evidence-based practice; KMS: knowledge management system.

Study	Setting/Type	Main Contribution	Key Finding for This Review	Orthopedic Relevance
Pereira and Fernandes (2025) [2]	Systematic review, health organizations	Maps KM tools and thematic clusters	Technology must be combined with human-resource and social-network dimensions	Supports a broad socio-technical view
Stoumpos et al. (2024) [21]	Bibliometric analysis	Maps KM and digital innovation in healthcare	Digital transformation and KM are converging rapidly	Useful for framing digital infrastructure
Hujala and Laihonen (2023) [22]	Qualitative case study, integrated care	Multi-level KM mechanisms	Macro, meso, and micro alignment is essential	Fits multi-site orthopedic care pathways
Brescia et al. (2025) [23]	Thematic analysis of academic and practitioner sources	AI and KM for sustainability and governance	Ethics, privacy, and implementation matter as much as automation	Relevant to AI adoption
Tajafari and Fanoodi (2025) [27]	SEM study, nurses	KM and evidence-based practice	KM improves EBP directly and indirectly	Transferable to orthopedic wards
Almashmoum et al. (2024) [29]	Concurrent mixed methods, cancer centers	Knowledge-sharing facilitators and barriers	Time pressure and weak incentives remain major barriers	Fits high-throughput orthopedic services
Hammarén et al. (2026) [32]	Cross-sectional study, managers	Management of digital competence sharing	Atmosphere is better developed than resource allocation	Relevant to service-line leadership
Vogt et al. (2026) [33]	Qualitative study, nursing practice	Expectations of an embedded KMS	Knowledge must fit point-of-care workflow	Directly relevant to orthopedic ward design
Karsikas et al. (2025; 2025) [30] [31]	Cross-sectional managerial studies	KM competence and implementation	Resources and systematic competence mapping are often inadequate	Strongly relevant to specialized hospital services
Gonçalves et al. (2024) [24]	Mixed methods, healthcare organizations	KMS, knowledge sharing, culture, quality	Technology and human-sharing routes can both support quality	Explains different orthopedic improvement pathways

Analytical Approach

An iterative and thematic approach was employed for data synthesis. Data extraction systematically captured information regarding study settings, methodological designs, specific KM focus, applied technologies or tools, reported mechanisms, clinical or organizational outcomes, identified barriers, and practical implications for orthopedic hospitals. For interpretive clarity, included sources were also read in three broad categories: orthopedic-specific, transferable general healthcare, and theoretical/policy/registry literature. The synthesized findings were subsequently categorized into six core themes, including strategic governance, knowledge capture and codification, multidisciplinary sharing, evidence use and pathway standardization, technology-enabled KM, and implementation barriers and enablers. Given the heterogeneity of the included evidence in terms of study design, setting, and evidentiary purpose, the use of a single standardized risk-of-bias tool was not considered appropriate across all sources. Instead, the included literature was appraised interpretively with attention to methodological design, publication recency, relevance to orthopedic hospital practice, contextual transferability, and conceptual contribution. These considerations informed the weighting and interpretation of the evidence throughout the review. Accordingly, the narrative synthesis prioritizes thematic convergence, explanatory value, and practice-relevant insights over formal comparison of quantitative effect estimates. Table 2 summarizes the comprehensive search strategy, eligibility logic, and analytical focus utilized to structure this review.

**Table 2:** Search strategy, eligibility logic, and analytical focus MEDLINE: Medical Literature Analysis and Retrieval System Online; Embase: Excerpta Medica database; CINAHL: Cumulative Index to Nursing and Allied Health Literature; KM: knowledge management; PROMs: patient-reported outcome measures; MDT: multidisciplinary team.

Component	Review Specification	Orthopedic Hospital Relevance	Implication for Synthesis
Databases	PubMed/MEDLINE, Scopus, Web of Science, Embase, CINAHL, Google Scholar	Captures clinical, managerial, nursing, and digital health evidence	Broad enough for a structured narrative review
Core Years	2020-March 2026, plus seminal earlier references	Emphasizes recent KM, digital, and implementation work	Balances recency with theoretical depth
Inclusion Focus	KM, knowledge sharing, evidence-based practice, registries, PROMs, digital systems, MDT coordination, patient safety, including direct orthopedic-specific and transferable hospital-based evidence	Matches the main knowledge flows in orthopedic hospitals	Allows direct and transferable evidence
Exclusion Focus	Irrelevant specialties, pure technical studies without organizational relevance, narrow-scope papers, opinion pieces	Reduces noise from nontransferable material	Improves thematic coherence
Analytical Themes	Governance; capture/codification; sharing; evidence use; digital infrastructure; barriers and enablers	Reflects distinctive orthopedic workflows	Supports actionable conclusions

Conceptualizing knowledge management in orthopedic hospitals

KM is often defined as the organized process through which institutions create, store, share, and apply knowledge to achieve goals [3,4,52-54]. In hospitals, this definition needs expansion. Clinical knowledge is not only a repository of guidelines and documents. It also includes tacit procedural expertise, sense-making under uncertainty, outcome data, near-miss learning, device experience, patient narratives, and coordination routines [1,3,4,52-54]. Orthopedic hospitals are therefore socio-technical systems in which knowledge exists in people, teams, documents, technologies, and workflows [2,3,23,46,55-58].

Classical KM theories remain constructive. Nonaka and Takeuchi’s SECI (Socialization, Externalization, Combination, and Internalization) model clarifies movement between tacit and explicit knowledge [52,53]. The knowledge-based view explains why organizational performance depends on the ability to integrate difficult-to-imitate knowledge resources [55]. Literature on dynamic capabilities helps explain why hospitals need to continually reconfigure knowledge assets in response to new evidence, technologies, and regulatory demands [55-58]. In healthcare, these perspectives intersect with evidence-based practice, quality improvement, implementation science, and learning health systems [59-66]. In this review, evidence-based practice is treated as the use of the best available evidence in clinical decision-making, and knowledge translation as the process of moving such evidence into routine care, whereas KM is used as the broader organizational framework that also includes knowledge creation, storage, sharing, and reuse. Recent cross-cutting healthcare KM literature supports this integrated reading because it repeatedly links KM with digital transformation, innovation, competence development, and evidence use [2,21-24,27,30-33].

Orthopedic hospitals add distinctive content to these theories. Much valuable knowledge is procedural and embodied: reduction technique, implant choice, soft-tissue handling, blood-loss anticipation, rehabilitation timing, or recognition of subtle postoperative risks. Some of this knowledge can be codified in pathways, operative notes, dashboards, and checklists, but some remains tacit and is shared through mentoring, case discussion, simulation, and team familiarity. This creates the central KM challenge in orthopedics, that is, hospitals must combine standardization with adaptive expert judgment [34-38,67].

**Figure 2:**
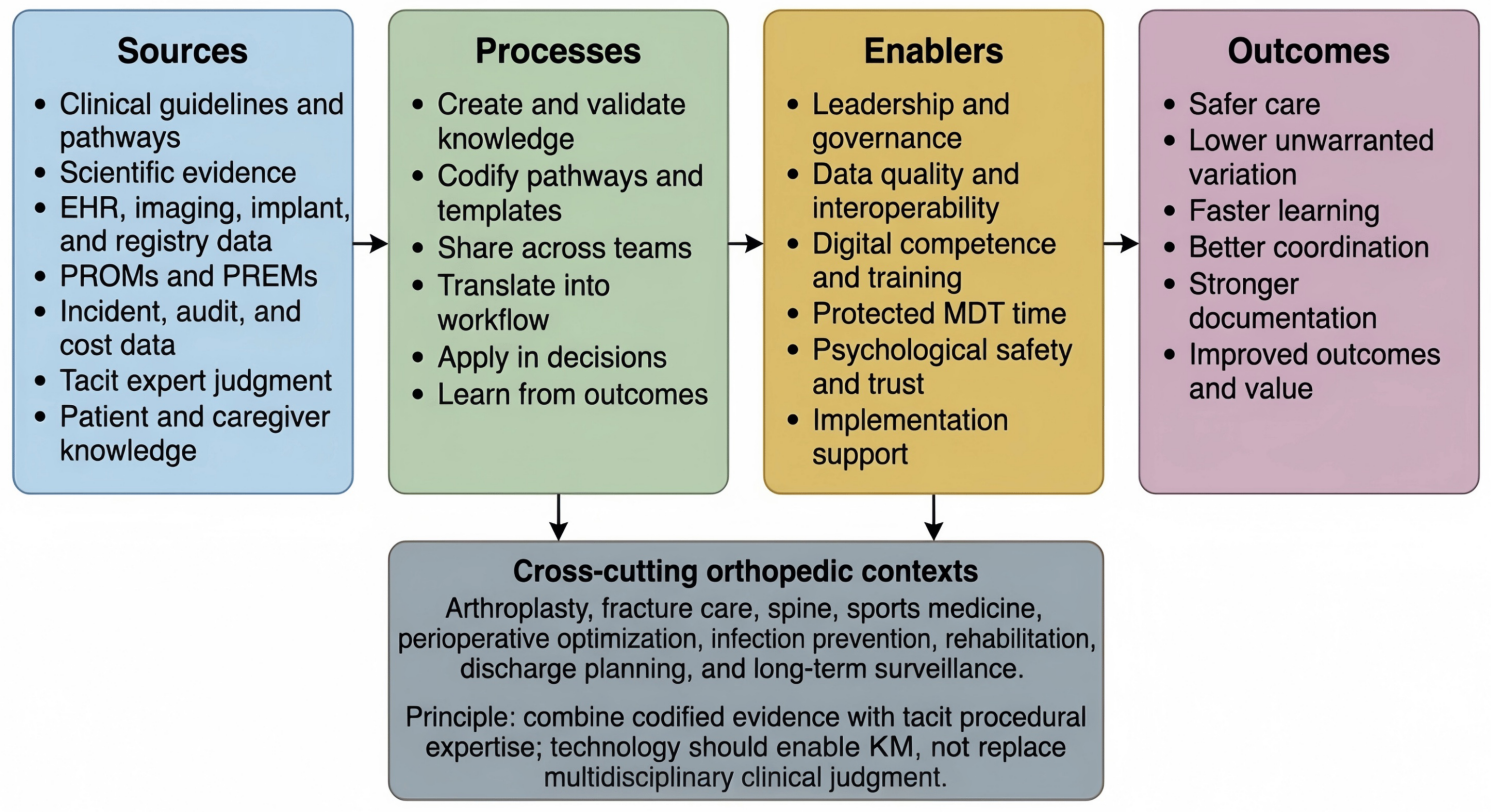
Integrated KM framework for orthopedic hospitals Original figure created with PowerPoint software (Microsoft Corporation, Redmond, Washington). EHR: electronic health record; PROMs: patient-reported outcome measures; PREMs: patient-reported experience measures; MDT: multidisciplinary team; KM: knowledge management.

Based on the reviewed literature, we derived a conceptual orthopedic KM model with four layers [1,3,4,52-55]. The first layer is knowledge sources, including guidelines, scientific evidence, registries, imaging, electronic health records (EHRs), implant vendor data, complication reviews, and patient-reported outcomes [5,6,10-14,39-42]. The second layer is KM processes encompassing creation, validation, codification, sharing, translation into local workflows, and reuse [1-4]. The third layer is enabling conditions, including leadership, culture, digital infrastructure, data quality, interprofessional trust, and protected learning time [15,22,30-33,68-73]. The fourth layer is outcomes, including patient safety, reduced variation, better coordination, faster learning, stronger documentation, staff competence, and more efficient service delivery [15,24-29,46,70-73]. Figure 2 synthesizes this model by linking knowledge sources, KM processes, enabling conditions, and outcomes while foregrounding the cross-cutting orthopedic contexts in which they interact.

This framework also explains why narrow technology adoption often underperforms [23,24,28,30,33]. Installing a dashboard or AI module does not by itself create organizational learning [7,23,24,46-51]. Digital tools work only when they fit clinical workflow, are trusted, produce interpretable outputs, and are connected to accountability and improvement routines [31-33,51,68,69,74,75]. Several recent healthcare KM studies converge on this point [23,24,29-33]. While technological infrastructure is essential, professional networks, effective leadership, continuous training, and competency development are of equal importance [25-27,30-33,70-73].

Strategic governance and organizational infrastructure

Contemporary literature increasingly frames KM as a core governance imperative rather than a peripheral administrative activity [2,15,21-25,28,30,31]. Within integrated care systems and hospital environments, knowledge should be systematically organized through defined roles, established routines, and robust infrastructures that facilitate its strategic application [22,28,30,76,77]. Recent investigations involving health and social care managers reveal a paradox: while leaders frequently evaluate their KM competencies favorably, particularly regarding the cultivation of a supportive organizational culture, they concurrently report deficiencies in planning competence development, allocating adequate resources, and systematically mapping the existing knowledge base of their workforce [30-32]. This discrepancy is exceptionally pertinent to orthopedic hospitals, where highly specialized medical care routinely encounters acute interdisciplinary coordination demands [22,30-32].

Orthopedic hospitals often benefit from formal KM governance frameworks given their management of high-risk and high-cost clinical processes [5,6,8,9,14,40-42,78-81]. For instance, arthroplasty programs commonly involve implant traceability, infection surveillance, revision analysis, and PROM feedback [5,6,14,40-42]. Similarly, trauma services typically rely on clinical handovers, rapid radiological interpretation, defined escalation pathways, and coordination among emergency, anesthesiology, and rehabilitation teams [16-18,43,80-82]. Furthermore, spine and sports medicine services frequently bridge inpatient and outpatient care settings. The literature suggests that, without sufficiently explicit governance structures, critical knowledge may remain confined within localized departmental silos or restricted to the tacit memory of experienced practitioners [15,22,30,31].

Several fundamental components of KM governance consistently emerge throughout the literature. The first is knowledge stewardship, which dictates that designated entities or individuals shall take ownership of the clinical knowledge architecture; this entails responsibility for guideline curation, clinical pathway maintenance, audit feedback mechanisms, and continuous learning from adverse complications [22,30,64,65]. The second component is data governance; healthcare institutions require stringent standards for data completeness, standardized terminology, seamless registry linkage, and secure access rights [5,6,14,40-42,46]. A third pillar is competence governance, necessitating that organizations systematically map existing expertise to identify where it is concentrated, where it is vulnerable to attrition, and how incoming personnel are integrated into established learning networks [30-32]. Finally, a robust review infrastructure is essential. When intentionally designed, morbidity and mortality conferences, implant review boards, infection case discussions, and multidisciplinary ward rounds all function as critical KM platforms [16,18,43,70-73].

Recent empirical studies by Hujala and Laihonen, Karsikas et al., and Hammarén et al. provide highly relevant insights in this context [22,30-32]. Collectively, these works demonstrate that effective KM is contingent upon the integration of macro-level structures, meso-level strategic initiatives, and micro-level clinical practices. When applied to orthopedic hospitals, this paradigm implies that national registries and regulatory frameworks, hospital-level strategies and data analytics, and bedside multidisciplinary interactions must be seamlessly aligned [5,6,14,22,30-32]. Crucially, sophisticated digital systems cannot compensate for a void in governance, just as a supportive organizational culture cannot fully mitigate the impact of inadequate resource allocation [15,24,25,30-32]. Consequently, the governance challenge in this domain is inherently both structural and relational [15,22,30-32].

A central implication of these findings is that orthopedic hospitals must transcend broad, abstract managerial discourse when addressing KM [22,30,31,78,79]. Instead, institutions should proactively establish a comprehensive KM portfolio encompassing registry intelligence, EHR configuration, competency mapping, and pathway governance, functioning alongside incident learning systems, patient information stewardship, and digital decision-support mechanisms [5-7,14,40-42,46-51]. Hospitals equipped with such an integrated portfolio are demonstrably better positioned to translate raw data into coordinated, actionable clinical interventions [15,24,30-33,46].

Knowledge capture, codification, and organizational memory

Knowledge capture encompasses the processes through which hospitals preserve clinically relevant experiences in formats suitable for future application [1,3,4,28,83]. Within orthopedic hospitals, organizational memory relies upon a diverse array of artifacts, including operative reports, implant logs, imaging archives, discharge summaries, rehabilitation protocols, ward dashboards, clinical checklists, registry submissions, incident reports, infection surveillance data, and PROM platforms. Each artifact encapsulates a distinct dimension of clinical practice [5,6,10-14,39-42,83]. Consequently, the fundamental KM challenge lies not merely in data collection, but in the seamless integration of these disparate sources [2,21-24,28,46,83].

Current literature indicates that codified knowledge most effectively enhances patient care when it is clinically meaningful, readily retrievable, and directly linked to decision-making processes [2,28,33,46,83]. EHR systems, clinical decision support tools, and digital documentation platforms can significantly increase access to explicit knowledge, mitigate information attrition, and facilitate evidence-based workflows [33,46,83]. Nevertheless, healthcare studies frequently reveal that frontline staff experience these documentation systems as fragmented, duplicative, or incongruent with actual clinical workflows [29,32,33,83]. A recent qualitative investigation into nurses’ expectations of a KMS is particularly illustrative, demonstrating that practitioners strongly prefer evidence resources to be integrated directly into care plans and patient charts, rather than relegated to separate digital repositories [33]. This insight is highly transferable to orthopedics. A clinical pathway or protocol situated outside the primary surgical workflow is inherently less effective than one organically embedded within preoperative assessments, clinical order sets, intraoperative documentation, and discharge planning [33,46,67-69].

Furthermore, orthopedic hospitals necessitate particularly robust organizational memory because clinical outcomes often unfold over protracted periods, spanning months or even years [5,6,14,40]. In this context, joint registries, infection surveillance mechanisms, implant tracking systems, and PROMs are instrumental in translating delayed outcomes into actionable knowledge [5,6,10-14,39-42]. National joint registries assume paramount importance; they transform individual surgical procedures into collective institutional learning by systematically tracking device performance, revision trajectories, surgeon-level variations, and broader institutional outcomes [5,6,14,40,41]. When such registry intelligence is synergized with localized clinical audits and case reviews, hospitals can successfully transition from passive data reporting to proactive KM [5,6,14,40-42].

However, the codification of knowledge has inherent limitations. Not all orthopedic expertise is reducible to standardized fields within an EHR or discrete steps in a clinical pathway [34-38,67,83]. Surgical judgment frequently relies heavily on pattern recognition, tactile feedback, and dynamic adaptation to unique anatomical or soft-tissue variations. The literature concerning orthopedic decision-making indicates that surgeons often prioritize experiential knowledge over formal empirical evidence, particularly under conditions of clinical uncertainty [34,35,37,38]. This tendency should not be misconstrued merely as a resistance to scientific evidence; rather, it often reflects a practical mismatch between generalized codified guidelines and the nuances of specific patient cases [34,37,38,67]. Effective KM, therefore, demands a dynamic and structured conversion between tacit and explicit knowledge [1,52,53]. Practices such as case-based debriefings, annotated imaging libraries, surgical video reviews, clinical mentoring, and collaborative audits are indispensable to facilitating this critical conversion process [16,17,38,67].

To strengthen knowledge capture, orthopedic hospitals can implement four actionable strategies [5,6,10-14,39,83]. First, clinical documentation should be redesigned to prioritize knowledge reuse rather than serving solely as a tool for legal and administrative compliance [33,46,83]. Second, institutions should seamlessly integrate registry, PROM, and EHR data at the specific service-line level [5,6,10-14,39-42]. Third, hospitals need to establish structured post-case reflection protocols to systematically analyze complications, surgical revisions, and unexpected clinical events [16,18,43,67,70-73]. Fourth, tacit expertise must be deliberately preserved and transferred through structured fellowship teaching, intraoperative coaching, and multidisciplinary case conferences [16,34-36,38,44,67]. While these may appear to be standard hospital routines, from a strategic KM perspective, they constitute the essential backbone of institutional organizational memory [1,3,4,83].

Knowledge sharing and multidisciplinary coordination

Knowledge sharing represents the most visible KM process within hospital settings, as patient care is fundamentally a collaborative endeavor [1,2,19,20,24,26,44]. In orthopedic hospitals, every major clinical pathway relies on distributed expertise. For instance, fracture management necessitates seamless continuity across emergency assessment, radiological imaging, surgical intervention, anesthesia, pain management, thromboprophylaxis, infection prevention, early mobilization, rehabilitation, and discharge planning. Similarly, arthroplasty requires meticulous perioperative optimization, precise implant selection, rigorous nursing surveillance, targeted physiotherapy, and longitudinal outcome monitoring. Sports medicine and spine services likewise depend on intricate coordination among surgeons, radiologists, pain management teams, therapists, and outpatient follow-up personnel. Across all these clinical settings, suboptimal knowledge dissemination inevitably precipitates operational friction and clinical risk [16-20,43-45].

Recent healthcare literature identifies consistent facilitators of and barriers to effective knowledge sharing. While frontline staff generally recognize the value of knowledge exchange and its direct correlation with patient safety, they frequently cite time constraints, staffing shortages, inadequate incentive structures, and fragmented communication platforms as significant impediments [18,25,29,32,44]. Mixed-methods research conducted in medical imaging departments revealed that face-to-face communication and email remain the predominant channels, whereas extrinsic motivation for proactive sharing is often lacking [29]. This finding is particularly consequential for orthopedic hospitals, where high case volumes and demanding operating room schedules severely restrict opportunities for clinical reflection. Taken together, these studies suggest that knowledge sharing is more likely to be sustained when supported by routine organizational structures rather than individual goodwill alone [16-20,29,43,44].

Multidisciplinary team meetings, morning trauma conferences, enhanced recovery huddles, infection control boards, and dedicated discharge rounds serve as pivotal mechanisms for knowledge exchange [16,18-20,43,44]. Crucially, their function extends beyond mere administrative routine; they actively synthesize isolated clinical observations into a cohesive, shared clinical understanding [16,19,20,44]. Recent investigations into multidisciplinary team (MDT) communication within acute care and surgical environments indicate that operational effectiveness is contingent upon clearly delineated roles, rapid access to pertinent information, and the cultivation of psychological safety [18-20,43]. In orthopedic hospitals, the absence of these foundational conditions can result in inconsistent mobilization plans, omitted anticoagulation protocols, inadequate pain management, or the delayed recognition of postoperative clinical deterioration [16-20,43].

Interprofessional KM inherently necessitates boundary-spanning knowledge translation. Surgeons and nursing staff frequently operate under disparate temporal rhythms and prioritize different informational cues [18-20,43,44]. For example, physiotherapists and case managers may identify potential discharge risks much earlier in the care continuum than surgeons typically appreciate. Similarly, clinical registry analysts might detect outcome signals that remain invisible to frontline teams unless the data are translated into actionable, practice-oriented language. Effective orthopedic KM, therefore, relies heavily on knowledge translators and brokers such as nurse leaders, pathway coordinators, quality officers, advanced practice providers, data analysts, and service-line managers, who are adept at navigating and bridging these professional boundaries [5,6,14,18-20,22,31,32,43-45].

The dissemination of tacit knowledge warrants specific emphasis. Senior surgeons routinely transmit invaluable clinical judgment through intraoperative mentoring, collaborative image review, complication case discussions, and informal corridor consultations [19,20,34-36,44]. Concurrently, experienced ward nurses possess critical tacit knowledge regarding early warning signs of patient deterioration, workflow bottlenecks, and specific gaps in patient education [19,20,44]. Hospitals that experience the attrition of such expertise through staff turnover without deliberate knowledge-transfer mechanisms may retain their formal documentation but still suffer a profound loss of institutional knowledge [1,3,4,44,52,53]. Recent KM studies focusing on system adoption, user expectations, and multidisciplinary knowledge sharing universally converge on a singular lesson: successful implementation occurs not merely because a technological system is deployed, but because healthcare professionals actively shape, interpret, and place trust in it during everyday practice [23,24,29,32,33,51,67-69,74,75]. Orthopedic hospitals are subject to this exact reality. Effective knowledge sharing is fundamentally a social endeavor before it becomes a digital one [19,20,24,29,43,44].

A paramount practical implication is that hospital KM strategies must approach multidisciplinary coordination as a primary clinical asset [16,18-20,43,44]. Shared visual boards, standardized clinical handovers, role-specific digital dashboards, daily interdisciplinary briefings, and structured debriefs are not adjuncts to knowledge management; rather, they constitute its very operational manifestation [16-20,43,44].

Knowledge management and evidence-based decision-making in orthopedics

A compelling rationale for a specialty-specific review lies in the sometimes observed gap between empirical evidence and real-world clinical decision-making within orthopedics [34,37,38]. Recent mixed-methods research concerning orthopedic practice variations suggests that formal, codified evidence may exert less influence on some daily clinical decisions than traditionally anticipated [34,37]. Instead, surgeons systematically synthesize trial evidence and clinical guidelines with experiential knowledge, peer influence, localized institutional norms, device familiarity, acute service pressures, and patient expectations [34,35,37,38]. This should not be interpreted as meaning that orthopedics is uniquely non-evidence-based or that evidence-based practice is absent; rather, it suggests that evidence is often incorporated alongside other forms of clinically relevant knowledge. From a KM perspective, this pattern highlights the value of frameworks that can accommodate how clinical knowledge is practically applied in the field [34,37,38,67].

Recent nursing-focused KM research provides an instructive parallel in this context [27,70-73,84,85]. For instance, Tajafari and Fanoodi demonstrated that effective KM can enhance evidence-based practice directly, as well as indirectly by shaping professional knowledge and attitudes [27]. A parallel logic is highly applicable to orthopedic hospitals. Clinical evidence is significantly more likely to drive decision-making when institutions establish accessible knowledge systems, foster positive professional attitudes toward evidence utilization, and seamlessly integrate this evidence into clinical workflows, rather than relegating it to supplementary reading [27,68-73,84,85]. Within orthopedic services, this functional integration may manifest as guideline-linked order sets, revision-risk digital dashboards, structured shared decision-making aids, and procedure-specific pathway prompts [10,34,38,46,67-69].

Clinical standardization proves exceptionally valuable in domains characterized by robust empirical evidence and high costs associated with unwarranted variation [64,65,80-82]. Prominent examples include thromboprophylaxis protocols, surgical site infection prevention bundles, enhanced recovery pathways, patient blood management, fracture discharge planning, and systematic PROM collection [42,80-82]. Nevertheless, such standardization requires careful calibration. A substantial proportion of orthopedic cases are complicated by patient comorbidities, frailty, surgical revision complexity, trauma severity, or competing therapeutic goals. Accordingly, this review interprets effective KM as supporting informed flexibility as well as standardization [34]. It may help standardize default practices while ensuring that clinical rationales for deviation remain transparent and open to multidisciplinary discussion. In KM terminology, this can be understood as harmonizing explicit clinical rules with structured, reflective review [34,37,38,67].

The implementation of PROMs aptly illustrates this operational challenge. Research within orthopedic clinics demonstrates that PROM collection is most efficacious when organically embedded into a multimodal clinical microsystem, rather than administered as an isolated, purely administrative survey exercise [10]. PROMs function as genuine KM tools only when the collected data are fed back to clinicians in highly interpretable formats, thereby directly influencing patient consultations, tailoring rehabilitation plans, or driving service-level redesign [10-13,39]. A corresponding principle applies to clinical registry data. Mere participation in a registry does not inherently elevate the quality of care. Measurable improvement is realized only when registry outcomes are actively discussed, rigorously benchmarked, and explicitly linked to actionable clinical initiatives [5,6,14,40-42].

Clinical decision support represents another domain of exponential growth [7,23,47-51]. AI and advanced analytics within orthopedics offer the promise of enhanced risk prediction, refined implant surveillance, automated imaging interpretation, and optimized perioperative planning [7,49,50]. While recent reviews validate this genuine potential, they simultaneously highlight critical concerns regarding algorithmic explainability, inherent bias, data governance, and overall workflow integration [7,49-51]. From a KM perspective, the fundamental challenge is not merely whether an algorithm can accurately predict a clinical outcome. The crux of the matter lies in whether that predictive insight can be trusted, appropriately contextualized, and effectively acted upon by multidisciplinary teams [23,24,32,51]. As a result, orthopedic hospitals need to conceptualize and evaluate AI as a sophisticated mechanism for knowledge augmentation, rather than a substitute for autonomous clinical decision-making [7,47,49-51].

Synthesizing these insights, the literature suggests that evidence-based decision-making in orthopedics may be strengthened when hospitals effectively manage four critical knowledge interfaces, including evidence-to-pathway translation, data-to-feedback loops, tacit-to-explicit knowledge exchange, and algorithm-to-clinician interpretation [5,6,10,14,34,37,38,46,51,67]. On this basis, KM may be considered an important component of a broader orthopedic quality strategy [15,68,69,78,79].

Digital infrastructure: EHRs, registries, dashboards, telehealth, and AI

Digital infrastructure now plays a major role in shaping the dissemination and flow of knowledge within modern hospitals [2,21,23,32,33,46-48]. Recent healthcare KM reviews identify digital platforms, EHRs, telemedicine, AI, Internet of Things, and clinical decision support systems as pivotal KM tools [2,21,23]. Within orthopedic hospitals, these technologies appear to have substantial practical relevance, given that orthopedic care is inherently data-rich and longitudinal [5-7,14,49,50]. In current practice, more established systems such as EHRs, registries, and dashboards often provide the core digital infrastructure, whereas AI and some advanced analytics may represent a more emergent or future-facing layer. Critical clinical data, spanning radiological imaging, implant specifications, laboratory values, intraoperative details, rehabilitation progress notes, and patient-reported outcomes, need to be seamlessly integrated to inform holistic care [5,6,14,40-42,46].

EHRs serve as the foundational substrate for this digital ecosystem. They possess the capacity to support knowledge continuity across diverse clinical settings, provided that their documentation templates, data fields, and information retrieval pathways accurately reflect operational clinical realities [33,46,83]. For orthopedic services, an optimized EHR architecture incorporates structured operative notes, precise implant data capture mechanisms, standardized postoperative milestones, organically embedded links to clinical evidence, specific rehabilitation trajectories, and highly visible risk stratification flags [33,46,83]. When clinical documentation is overly generic, the system functions merely as an information repository rather than an active facilitator of knowledge application [83]. Consequently, KMS design studies within the nursing discipline consistently emphasize the necessity of process integration and end-user usability [33]. These principles are also likely to be relevant in orthopedic settings [33,46,83].

Clinical registries represent a preeminent KM asset specific to the orthopedic specialty [5,6,14,40,41]. National joint registries and specialized service-line databases establish robust feedback loops that individual hospitals cannot cultivate independently. They illuminate longitudinal trends regarding complication patterns, implant failure trajectories, readmission frequencies, and surgical revision rates [5,6,14,40,41]. Critical evaluations of major joint registries underscore their indispensable role in enhancing patient outcomes through continuous surveillance and rigorous benchmarking. However, these studies concurrently highlight the prerequisite for high-fidelity data, transparent governance structures, and meaningful localized interpretation [5,6,14,40,41]. While registry-fed digital dashboards empower clinical services to identify performance outliers, benchmark against peer units, and target quality improvement initiatives, the true epistemic value is derived from structured multidisciplinary interpretation routines, rather than from the digital dashboards in isolation [5,6,14,39-42].

Telehealth and remote monitoring platforms appear to play a growing role in modern orthopedic care. Although orthopedic KM is frequently associated primarily with surgeons and operating theaters, sustaining knowledge continuity post-discharge is also clinically important [10-14,39]. Digital follow-up platforms, telerehabilitation programs, integrated patient portals, and remote PROM capture mechanisms may help sustain information flow across the hospital-community boundary [10-14,39,46]. Evidence derived from the broader telehealth literature suggests that hybrid care models may help alleviate system burdens and support care continuity, while preserving the capacity for in-person clinical assessments when medically indicated [46]. For orthopedic hospitals, such remote modalities may be useful for tracking postoperative symptomatology, monitoring rehabilitation adherence, conducting wound surveillance, and delivering patient education [10-13,39,86].

AI constitutes the most rapidly advancing technological domain within this landscape [7,23,47-51]. Systematic reviews evaluating AI in orthopedic research and surgical decision-making describe promising applications in automated image analysis, predictive risk stratification, precision implant planning, and prognostic modeling [7,49,50]. However, these applications should be distinguished from mature routine implementation. In many settings, AI appears to remain at an early, selective, or evaluative stage rather than a fully established component of everyday orthopedic KM practice. The deployment of AI therefore illustrates the critical dichotomy between mere data abundance and actionable clinical knowledge. Algorithmic models necessitate rigorously curated training datasets, robust clinical validation, stringent governance, and seamless integration into established decision-making workflows [7,47-51]. In the absence of these prerequisites, AI systems risk generating clinical noise, fostering unwarranted provider overconfidence, or perpetuating hidden systemic biases [7,49-51]. Recent healthcare literature intersecting AI and KM argues that ethical governance, privacy protections, and human-centered implementation are inseparable from technical performance [23,24,51]. Accordingly, AI may be best viewed as a developing adjunct with future potential, rather than as a mature substitute for established clinical knowledge systems. Orthopedic hospitals may therefore benefit from a stratified digital strategy comprising reliable core EHRs, seamless registry linkages, highly interpretable dashboards, selective AI augmentation, and clear frameworks for clinical accountability [7,23,32,46-51].

Digital competence represents the essential human dimension of this technological infrastructure. Studies examining managers’ self-assessed leadership regarding digital competence sharing reveal that a supportive organizational atmosphere, while necessary, is insufficient; dedicated time, adequate resource allocation, and continuous training remain persistent vulnerabilities [31,32]. Accordingly, orthopedic hospitals need to conceptualize and cultivate digital literacy as a core KM capability [31-33]. While clinical staff are not required to function as data scientists, they must possess a foundational understanding of what information the system archives, how data fidelity directly influences clinical decision-making, how performance dashboards should be accurately interpreted, and under what circumstances algorithmic outputs should be critically questioned [31-33,51].

Finally, the patient-facing dimension of KM warrants distinct emphasis [10-13,39,86]. In orthopedics, patients navigate a complex continuum traversing initial outpatient consultations, surgical interventions, acute inpatient recovery, home-based rehabilitation, and frequently, long-term revision surveillance [5,6,10-14,39-42,86]. At each juncture, patients assimilate knowledge through diverse modalities, including informed consent discussions, structured preoperative education, formal discharge instructions, tailored rehabilitation plans, PROM questionnaires, and digital follow-up communications. When these informational materials are inconsistent, chronologically outdated, or cognitively disconnected from the primary clinical workflows, hospitals forfeit a critical opportunity to promote safer patient self-management and enhance care continuity [10-13,39,86]. Conversely, when patient-facing information is systematically curated as an integral component of the overarching KM architecture alongside clinician-facing tools, hospitals can effectively align patient education with standardized clinical pathways, robustly reinforce shared decision-making, and empower patients to seamlessly recognize and escalate postoperative concerns [10-13,39,46]. This alignment is especially consequential for arthroplasty and fracture care, where patient adherence to wound care directives, multimodal analgesia regimens, early mobilization guidance, and follow-up protocols directly impacts complication rates, readmission metrics, and overall patient experience [42,80-82,86]. In this context, patient education is not a peripheral adjunct to KM. Rather, it serves as a primary mechanism through which institutional organizational knowledge is rendered clinically actionable beyond the hospital walls [10-14,39-42,86].

KM, patient safety, quality, and performance in orthopedic hospitals

The primary rationale for implementing KM transcends mere administrative efficiency. It is fundamentally anchored in patient safety and clinical performance [14,15,24,68,69]. The synthesized literature suggests several plausible pathways through which KM may contribute to safer care and better organizational performance in orthopedic hospitals, although direct orthopedic evidence remains limited [2,5-7,10-18,21-33,37-43,46-51,67,83].

First, KM effectively mitigates care fragmentation. Enhanced knowledge continuity across the preoperative, intraoperative, and postoperative phases significantly diminishes the risks of clinical omissions, contradictory instructions, and delayed therapeutic responses [10-13,16,17,27,39]. This continuity is especially critical in managing thromboprophylaxis, antimicrobial stewardship, patient blood management, discharge planning, multimodal pain control, and rehabilitation sequencing [18,42,43,80-82]. Second, KM facilitates rigorous clinical standardization in domains where practice variation yields no clinical benefit [34,42,64,65,80-82]. The deployment of evidence-based clinical pathways, digitally embedded order sets, and shared performance dashboards substantially improves care reliability for high-volume procedures, notably hip and knee arthroplasty [39,42,80-82]. Third, KM enables the proactive detection of adverse events through continuous registry signal monitoring, targeted infection surveillance, automated audit data analysis, and structured routine case reviews [5,6,14,40-42,80,81]. Fourth, KM significantly bolsters overarching staff capability [27,30-32,70-73,84,85]. While broader healthcare studies confirm that KM strongly correlates with elevated workforce performance, robust evidence-based practice, and superior service quality [24-29], there is compelling reason to anticipate commensurate benefits within orthopedic hospitals, provided that competency development is systematically structured [27,30-32,70-73,84,85].

The nexus between KM and patient safety is of paramount importance concerning systemic clinical risks [15,18,43,80-82,86]. Orthopedic hospitals routinely navigate complex challenges, including stringent implant traceability, perioperative hidden blood loss, postoperative surgical site infections, inpatient falls, venous thromboembolism (VTE), preventable hospital readmissions, delirium among geriatric fracture cohorts, and rehabilitation failures [5,6,8,9,14,40-42,80-82,86]. Mitigating each of these risks is inextricably linked to high-fidelity information quality and expeditious knowledge exchange [15-20,43]. For instance, managing hidden blood loss necessitates specialized training, rapid clinical recognition, meticulous documentation, and mutually established thresholds for intervention; however, recent investigations into hospital nurses’ knowledge and practice indicate that such expertise remains unevenly distributed [84,85]. Similarly, effective infection prevention demands rigorous bundle compliance, continuous surveillance, and active feedback loops [80-82], while comprehensive implant surveillance mandates consistent registry participation coupled with robust local peer review [5,6,14,40,41]. Ultimately, the successful execution of these safety measures is fundamentally KM-dependent [5,6,14-17,80-82].

Furthermore, organizational performance needs to be interpreted through a comprehensive lens. Beyond traditional clinical outcomes, orthopedic hospitals are profoundly invested in optimizing length of stay, maximizing operating theatre efficiency, enhancing staff retention, elevating the patient experience, minimizing readmissions, reducing revision burdens, and rationalizing resource utilization [5,6,8-13,39,42]. For example, enhanced short-stay arthroplasty models aptly illustrate how deep pathway knowledge, multidisciplinary coordination, and strict implementation discipline can generate substantial cost and workflow efficiencies without compromising patient safety, provided that patient selection and follow-up protocols are robustly maintained [42]. PROM systems introduce a vital performance dimension by quantifying patient experience and functional recovery in a highly structured format [10-13,39]. Here again, the core KM challenge extends beyond mere data collection; it lies in effectively transforming these raw metrics into actionable, service-level institutional learning [5,6,10-14,39-42].

The study by Gonçalves, Curado, and Oliveira is particularly salient in this context, despite not being exclusively orthopedic-specific [24]. Their mixed-methods findings posit that both advanced technological systems and proactive human knowledge-sharing behaviors independently and synergistically bolster the quality of care [24]. This dual-route insight elucidates why certain orthopedic hospitals achieve significant quality improvements with only modest digital sophistication provided they possess a robust teamwork and collaborative review culture, whereas others underperform despite deploying highly expensive information technology infrastructures [15,24]. In essence, while technology and human coordination can partially compensate for each other's deficits, neither is entirely substitutable. The most profound and sustainable results inevitably emerge from the strategic integration of both domains [15,24].

Overall, the literature supports a cautious practical interpretation. Orthopedic hospitals equipped with more developed KM ecosystems may be better positioned to mitigate systemic clinical risks and support patient safety performance [15,24,27,68-73]. However, much of this inference is based on plausible mechanisms and transferable findings from related healthcare contexts, rather than extensive direct orthopedic outcome studies [2,15-33,37,38,43-45,67-75,83-85].

Discussion

Barriers, Failure Modes, and Equity Considerations

This review additionally clarifies the underlying reasons why KM initiatives frequently fall short of expectations. The most pervasive barrier is systemic fragmentation [25,28]. Critical data are sequestered in siloed systems, clinical departments maintain disparate operational routines, and accountability for overcoming these integration challenges remains unassigned [23,24]. A second significant impediment is chronic time scarcity. Although frontline staff generally value continuous learning, they frequently lack protected time for in-depth case reviews, documentation optimization, formal training, or interdisciplinary communication [29,30]. A third barrier relates to misaligned incentive structures. While the sharing and codification of knowledge consistently yield collective organizational benefits, they often offer limited tangible rewards for the individual practitioner [25,29]. A fourth challenge is implementation overload. Hospitals frequently introduce digital dashboards, clinical checklists, care pathways, new registries, and AI pilot programs concurrently, often lacking the requisite strategic coordination or seamless workflow integration [23,24,32,33,68,69].

While organizational culture is undeniably critical, the literature cautions against relying on vague cultural determinants [15,24,87]. A nominally supportive culture is efficacious only when underpinned by robust operational structures. Several recent studies reveal a compelling discrepancy: administrative managers frequently rate institutional KM competence far more positively than frontline staff perceive the actual availability of supportive resources [30-32]. Orthopedic hospitals must be acutely aware of this perceptual mismatch. Leadership may presume that institutional learning is adequately supported, whereas clinical staff simultaneously experience chronic workflow interruptions, excessive documentation burdens, and constrained access to meaningful, actionable feedback [30-32]. Comprehensive KM diagnostics should thus systematically incorporate both managerial and frontline clinical perspectives [30-32].

Another prevalent failure mode is an overreliance on knowledge codification [1,3,4,52,53]. Institutions may operate under the misconception that the mere publication of a guideline or the uploading of a clinical protocol equates to effective knowledge management [1,64,65,83]. In practice, codified materials undergo rapid informational decay unless they are actively curated, clinically interpreted, and continuously reinforced through collaborative social processes [1-4,28,33,52,53]. Orthopedic practice is particularly vulnerable to this limitation, as the highly nuanced nature of procedural expertise and inherent case complexities often render simplistic rule transfers ineffective [34-38,67]. Conversely, hospitals risk overvaluing tacit knowledge [34,37,38]. When clinical decision-making relies excessively on entrenched local habits or the subjective preferences of senior practitioners, the utilization of empirical evidence and the capacity for organizational learning inevitably atrophy [34,37,38,67]. Robust KM frameworks successfully navigate between these two extremes, harmonizing codified guidelines with experiential insights [1,34,37,38,52,53,67].

Furthermore, the critical issues of digital and data inequity deserve explicit consideration [7,31,32,46-51]. Substantial disparities exist among hospitals regarding analytical capacity, clinical registry maturity, and systemic interoperability [5,6,14,31,32,46]. Intra-organizationally, healthcare professionals exhibit varying levels of digital competence and unequal access to structured performance feedback [31-33]. From a patient-facing perspective, an overreliance on digital follow-up and portal-based communications risks alienating specific demographic groups, particularly older adults, individuals with limited health literacy, or those lacking consistent access to digital devices [46,86]. Given that orthopedic hospitals frequently serve aging and vulnerable populations, the design of digital KM architectures should proactively incorporate accessible alternatives and robust user support mechanisms [46,86].

Finally, inherent limitations within the current research design landscape should be acknowledged [2,21-33]. A substantial proportion of the existing healthcare KM literature remains cross-sectional, primarily descriptive, or conceptually broad [2,21-33]. While these methodological approaches are highly useful for identifying underlying mechanisms, they inherently lack the statistical power required to substantiate definitive causal claims [2,21-33]. Therefore, orthopedic hospitals should interpret the current body of evidence as a compelling rationale for proactive intervention, coupled with a simultaneous mandate for more rigorous longitudinal evaluation [34,67-69,74,75]. The field has achieved sufficient maturity to transcend abstract endorsements of KM principles. However, it has not yet reached a state where every KM intervention can be assumed universally efficacious across all clinical contexts [67-69,74,75].

Implications for Practice and a Research Agenda

The evidence synthesized in this review supports a practical model for orthopedic hospitals [6,7,10,14,16,18,32,34,42,43,49,51,83]. Table 3 translates the practice implications into priority KM domains, mechanisms, barriers, and illustrative metrics specific to orthopedic care settings.

**Table 3:** Priority KM domains, mechanisms, barriers, and metrics for orthopedic hospitals Table Credits: Xiao Du, Mingyuan Zhao, and Zhuo Fu. This table was created by the authors and compiled from information derived from published sources [6,7,10,14,16,18,32,34,42,43,49,51,83]. It is an original synthesis and is not directly reproduced from any single previously published table. KM: knowledge management; MDT: multidisciplinary team; VTE: venous thromboembolism; PROM: patient-reported outcome measure; SSI: surgical site infection; AI: artificial intelligence.

KM Domain	Typical Mechanisms	Example Orthopedic Applications	Common Barriers	Illustrative Metrics
Governance	Service-line ownership, pathway committees, registry oversight	Arthroplasty governance board; fracture pathway steering group	Diffuse accountability	Pathway review frequency; action closure rate
Knowledge Capture	Structured notes, implant logs, dashboards, case review	Implant traceability; complication audit; imaging libraries	Poor data quality; duplicate documentation	Data completeness; audit turnaround time
Knowledge Sharing	Handover tools, MDT rounds, debriefs, mentoring	Trauma conference; ward-board rounds; operating room coaching	Time pressure; siloed teams	Handover reliability; staff-reported sharing climate
Evidence Translation	Guideline-linked order sets, decision aids, prompts	VTE prophylaxis bundle; infection prevention checklist; blood management protocol	Local variation; low trust in external evidence	Bundle adherence; unwarranted variation indicators
Learning from Outcomes	Registries, PROMs, infection surveillance, readmission review	Revision analysis; PROM dashboard; SSI surveillance	Delayed feedback; weak analytic capacity	PROM completion; revision trends; SSI rate
Digital Capability	Training, usability testing, dashboard literacy, AI governance	PROM portal use; risk prediction tool; telerehabilitation follow-up	Low digital competence; interoperability gaps	Training completion; dashboard access; AI override review

KM may be conceptualized as a foundational clinical infrastructure comprising five operational pillars [22,30-33,68,69]. The first pillar is an integrated knowledge architecture [5,6,10-14,22,30-32,39,46]. Hospitals may benefit from interlinking clinical guidelines, care pathways, EHR templates, digital dashboards, registries, and PROMs at the specific service-line level [5,6,10-14,39,46]. Fragmented systems may need strategic redesign to align with actual orthopedic workflows [33,46,83]. The second pillar entails multidisciplinary learning routines [16-20,43-45]. Daily clinical briefings, structured handovers, implant reviews, morbidity and mortality conferences, infection control boards, and discharge huddles can be treated as formalized knowledge-sharing practices rather than solely administrative meetings [16,18-20,43,44]. The third pillar focuses on workforce capability [30-32,70-73,84,85]. Management may consider systematically mapping clinical competencies, identifying critical single points of knowledge dependency, and supporting formal mentoring, simulation-based training, and digital skills development [30-32,70-73,84,85]. The fourth pillar centers on continuous feedback and benchmarking [5,6,11-14,39-42]. Registry intelligence, clinical audit data, and PROMs can be translated into actionable, recurring reports that are reviewed by teams with explicit accountability [5,6,14,40-42]. The fifth pillar is robust implementation governance [67-69,74,75]. The introduction of any novel digital tool or clinical pathway may be supported by clearly delineated ownership, workflow compatibility assessments, targeted training protocols, defined evaluation metrics, and scheduled review intervals [23,32,33,51,67-69,74,75].

Regarding future research directions, three priority areas may be highlighted. First, the development of orthopedic-specific KM measurement tools remains in its infancy [22,24,27,30-32]. Subsequent investigations could usefully examine composite indicators that synthesize pathway reliability, data completeness, case-review quality, PROM uptake, knowledge-sharing climate, and the practical utilization of outcome feedback [5,6,10-14,39-42]. Second, prospective implementation studies would be valuable [67-69,74,75]. Rather than merely surveying general staff attitudes toward knowledge sharing, research could empirically examine the operational impacts of integrating a registry dashboard into an arthroplasty review, embedding evidence links into fracture order sets, or systematically redesigning MDT communication architectures [5,6,10,14,16,17,40-42,67]. Third, comparative study designs may also be informative [15,24,30-32,68,69]. Given that orthopedic institutions employ highly variable configurations of digital tools, organizational culture, staffing models, and governance structures [7,15,24,30-33,46-51], comparative analyses of these diverse models would facilitate the identification of optimal, real-world configurations that drive high performance [15,24,30-33,68,69].

Furthermore, several subtle inquiries arise from the cross-cutting literature. Studies examining healthcare KM, digital systems, and implementation consistently underscore critical knowledge gaps at the intersection of technology, human behavior, and organizational context [7,23,24,49-51]. How do human incentive structures interact with KMS adoption within orthopedic wards [25,29]? Which dimensions of tacit surgical expertise can be effectively codified without compromising clinically vital nuances [30-33]? How should AI be optimally integrated into shared decision-making processes between surgeons and patients [49-51]? Which implementation strategies most effectively enhance knowledge utilization among trauma teams operating under acute time constraints [37,38]? How are registry-derived signals successfully translated into localized clinical action, and which organizational structures most effectively accelerate this translation [23,24,29,67]? These represent highly actionable, clinically grounded research imperatives rather than abstract theoretical exercises [67-69,74,75].

A final implication pertains to theoretical frameworks. Orthopedic KM research needs to avoid establishing false dichotomies among classical KM theory, implementation science, and the learning health system paradigm [3,4,52-58,60-66,68,69]. Instead, these frameworks should be synergistically integrated. KM provides the foundational vocabulary for knowledge creation, sharing, codification, and reuse [1,3,4,52-58]. Implementation science offers critical methodologies for understanding contextual adoption and operational barriers [60,61,66,68,69,74,75]. Meanwhile, the learning health systems model explains the mechanisms through which raw data are transformed into iterative clinical improvements [62,63,88]. Orthopedic hospitals inherently operate at the dynamic intersection of all three domains [2,5,6,14,34,46].

From an operational perspective, KM within orthopedic hospitals is most effectively implemented through a phased approach rather than a single massive transformational program [15,22,23,30-33,68,69]. A practical sequence begins with establishing service-line governance, defining pathway priorities, and selecting a concise set of clinically meaningful metrics. Following this foundation, hospitals can enhance knowledge capture through documentation redesign, registry linkages, and routine case reviews. Only after these steps are secure should institutions scale digital dashboards, predictive tools, or other advanced analytics [5,6,10-14,30-33,39-42,46]. This strategic sequencing is crucial because hospitals frequently prioritize early technological investments, only to later realize that unclear ownership, poor workflow integration, or inadequate data quality undermine long-term use [23,24,32,33,46,51,68,69]. Furthermore, the synthesized literature indicates that mature KM relies on cumulative organizational alignment. Executive leaders need to explicitly define responsibilities, while mid-level managers need to allocate dedicated time and build supportive feedback structures. Simultaneously, frontline clinical teams require tools that are intuitive enough to use under intense clinical pressure [15,22,30-33,68,69]. Orthopedic services are exceptionally well-suited to this staged approach because many clinical pathways already possess natural anchors for iterative learning cycles. These include morning trauma meetings, arthroplasty governance boards, infection control reviews, morbidity and mortality conferences, and structured postoperative rehabilitation checkpoints [5,6,14,16,18,40-43,67]. These established routines can serve as the organizational backbone upon which increasingly sophisticated KM capabilities are built [15,16,18,22,30-33,43,67].

Limitations

It is important to acknowledge several limitations of this review. First, this study was conducted as a structured narrative review rather than a full systematic review or meta-analysis. The included literature was methodologically diverse, spanning qualitative, quantitative, and mixed-methods studies as well as conceptual, policy, and registry-oriented sources. Accordingly, the evidence base was not considered suitable for uniform quantitative comparison or for the application of a single standardized risk-of-bias instrument across all source types. Rather, the review relied on a context-sensitive critical reading of each source, with judgments informed by its methodological characteristics, practical relevance, transferability to orthopedic hospital settings, and contribution to the overall explanatory framework. While this approach was appropriate for the review objective and for the heterogeneous nature of the evidence, it necessarily limits the precision of quantitative inference. In addition, although recent literature was deliberately prioritized, selected earlier foundational references were retained to provide theoretical and institutional context. Finally, although the search and screening process followed a transparent and structured approach, the resulting synthesis remains interpretive in nature. It emphasizes thematic convergence, explanatory value, and practice-relevant insights rather than exhaustive retrieval or pooled effect estimation. Accordingly, the framework presented in Figure 2 should be interpreted as an author-derived conceptual synthesis rather than an empirically validated model. The conclusions of this review should therefore be read as suggestive and conceptually integrative rather than conclusive estimates of effect.

## Conclusions

KM in orthopedic hospitals may be understood as the systematic integration of evidence, clinical experience, data, and teamwork across the care pathway. One important insight from the reviewed literature is that orthopedic performance relies on more than data volume alone. It appears to depend in part on how effectively hospitals connect codified evidence with tacit clinical knowledge and integrate that combined expertise into multidisciplinary workflows. Orthopedic hospitals have distinctive KM demands because they depend heavily on procedural expertise, continuous implant surveillance, structured outcome feedback, and coordinated rehabilitation. These characteristics make KM both challenging and potentially valuable.

Current evidence, including transferable findings from related healthcare settings, suggests that effective KM may support evidence-based practice, digital adoption, multidisciplinary coordination, and the mitigation of systemic clinical risks. However, these findings should be interpreted cautiously because direct orthopedic evidence remains limited and much of the synthesis is interpretive. Meaningful improvement is likely to depend on how leadership, workflow integration, competence development, and feedback processes are aligned with daily orthopedic realities. The future of this field would benefit from moving beyond broad theoretical advocacy toward more specialty-specific design and prospective evaluation. Orthopedic hospitals would benefit from KM models that are measurable, practically implementable, and aligned with patient safety and long-term clinical outcomes. When recognized and supported as a core element of clinical infrastructure, KM may offer a practical route toward safer care, reduced unwarranted variation, continuous organizational learning, and more reliable performance across orthopedics and traumatology.

## Appendices

**Table 4:** Full search strategy

Search Timing
Database searches were conducted on 23 March 2026 and last updated on 27 March 2026. The primary time window was January 2020 to March 2026, with selective inclusion of earlier seminal theoretical, registry, and policy references for contextual grounding.
Core Concept Blocks
1. Knowledge management terms:
“knowledge management” OR “knowledge sharing” OR “knowledge transfer” OR “knowledge mobilization” OR “knowledge translation” OR “organizational learning” OR “clinical decision support” OR “evidence-based practice”
2. Orthopedic terms:
“orthopedic” OR “orthopaedic” OR “trauma” OR “arthroplasty” OR “musculoskeletal” OR “joint replacement” OR “fracture”
3. Hospital-setting terms:
“hospital” OR “surgical ward” OR “operating room” OR “rehabilitation” OR “acute care”
4. Digital/organizational enablers:
“electronic health record” OR “registry” OR “dashboard” OR “PROM” OR “artificial intelligence” OR “implementation” OR “multidisciplinary team”
Representative Combined Strategy
(“knowledge management” OR “knowledge sharing” OR “knowledge transfer” OR “knowledge mobilization” OR “knowledge translation” OR “organizational learning” OR “clinical decision support” OR “evidence-based practice”)
AND
(“orthopedic” OR “orthopaedic” OR “trauma” OR “arthroplasty” OR “musculoskeletal” OR “joint replacement” OR “fracture”)
AND
(“hospital” OR “surgical ward” OR “operating room” OR “rehabilitation” OR “acute care”)
AND
(“electronic health record” OR “registry” OR “dashboard” OR “PROM” OR “artificial intelligence” OR “implementation” OR “multidisciplinary team”)
Databases Searched
PubMed/MEDLINE, Scopus, Web of Science, Embase, CINAHL, and Google Scholar. Searches were adapted to the syntax and field structure of each database.
